# 

**Published:** 1894-06

**Authors:** 


ESTABLISHED 1855. SUBSCRIPTION, $2.00.

### ATLANTA MEDICAL AND SURGICAL JOURNAL.

LIBRARY [illegible]

OLD SERIES, VOL. XXVI.

NEW SERIES, VOL. XI.

ATLANTA, GA., JUNE, 1894. No. 4.

LUTHER B. GRANDY, M.D., MANAGING EDITOR.

M. B. HUTCHINS, M.D., BUSINESS MANAGER.

